# The Mast Cell Degranulator Compound 48/80 Directly Activates Neurons

**DOI:** 10.1371/journal.pone.0052104

**Published:** 2012-12-18

**Authors:** Michael Schemann, Eva Maria Kugler, Sabine Buhner, Christopher Eastwood, Jemma Donovan, Wen Jiang, David Grundy

**Affiliations:** 1 Human Biology, Technische Universität München, Freising, Germany; 2 Department of Biomedical Sciences, University of Sheffield, Sheffield, United Kingdom; University of California, Los Angeles, United States of America

## Abstract

**Background:**

Compound 48/80 is widely used in animal and tissue models as a “selective” mast cell activator. With this study we demonstrate that compound 48/80 also directly activates enteric neurons and visceral afferents.

**Methodology/Principal Findings:**

We used in vivo recordings from extrinsic intestinal afferents together with Ca^++^ imaging from primary cultures of DRG and nodose neurons. Enteric neuronal activation was examined by Ca^++^ and voltage sensitive dye imaging in isolated gut preparations and primary cultures of enteric neurons. Intraluminal application of compound 48/80 evoked marked afferent firing which desensitized on subsequent administration. In egg albumen-sensitized animals, intraluminal antigen evoked a similar pattern of afferent activation which also desensitized on subsequent exposure to antigen. In cross-desensitization experiments prior administration of compound 48/80 failed to influence the mast cell mediated response. Application of 1 and 10 µg/ml compound 48/80 evoked spike discharge and Ca^++^ transients in enteric neurons. The same nerve activating effect was observed in primary cultures of DRG and nodose ganglion cells. Enteric neuron cultures were devoid of mast cells confirmed by negative staining for c-kit or toluidine blue. In addition, in cultured enteric neurons the excitatory action of compound 48/80 was preserved in the presence of histamine H_1_ and H_2_ antagonists. The mast cell stabilizer cromolyn attenuated compound 48/80 and nicotine evoked Ca^++^ transients in mast cell-free enteric neuron cultures.

**Conclusions/Significance:**

The results showed direct excitatory action of compound 48/80 on enteric neurons and visceral afferents. Therefore, functional changes measured in tissue or animal models may involve a mast cell independent effect of compound 48/80 and cromolyn.

## Introduction

Mast cells play a fundamental role for physiological as well as pathological functions [1], [2]. They contain a plethora of bioactive mediators which are released upon IgE-directed and non-IgE-directed stimulation. Compound 48/80 is a mixed polymer of p-methoxy-N-methyl phenylethylamine crosslinked by formaldehyde and widely used for non-IgE dependent stimulation of mast cells. It was first characterized as a histamine releaser in 1951 [3]. It is generally assumed that compound 48/80 specifically activates and degranulates mast cells. In line with this notion, injection of compound 48/80 evoked inflammatory responses in various animal models [4].

There are many reports that functional changes induced by application of compound 48/80 involve activation of nerves as a consequence of mast cell activation. The gut lamina propria contains numerous mast cells many of which are closely associated to nerves of the enteric nervous system (ENS) and visceral afferents [5]. Conditions associated with mast cell activation in the gut are among others visceral pain. Intraperitoneal administration of compound 48/80 leading to systemic mast cell degranulation may cause visceral pain [6]. The cascade leading to nociception is believed to start with mast cell activation, followed by mediator release and finally activation of nociceptors [6]. The same assumption has been made by direct application of compound 48/80 to isolated colon segments [7]. In this study it was reasoned that enhanced release of calcitonin gene-related peptide from visceral afferents is a consequence of compound 48/80 induced mast cell activation. Application of compound 48/80 to intact oesophagus preparations induced a longitudinal muscle contraction that was prevented by neural TRPV1 (transient receptor potential vanilloid 1) antagonism [8]. Again the conclusion was that neural activation is secondary to liberation of mast cell mediators. Studies on co-cultures of mast cells with dorsal root ganglion (DRG) cells or sympathetic neurons concluded that compound 48/80 induced mast cell stimulation resulted in activation of neurons [9], [10]. The interpretation of the above mentioned results and that of many other studies is based on the assumption that compound 48/80 selectively activates mast cells.

It is noteworthy that, whenever tested, compound 48/80 altered functions were inhibited or even abolished after blocking nerve activation. This is of course expected if the cell or tissue function is modulated by nerve activation that is initially triggered by mast cell mediator release. Nevertheless, one would assume that histamine release by compound 48/80 should have direct actions on histamine receptor expressing target cells. For example, one may raise the questions of why compound 48/80 induced longitudinal muscle contraction was almost prevented by TRPV1 antagonism although both muscle layers express functional histamine receptors [8].

Doubts on the specificity of compound 48/80 have been raised in the early literature [11], [12] but largely ignored in the absence of a systematic investigation of neural activation. Here we describe effects of compound 48/80 on both enteric neurons and visceral afferents that are independent of mast cell degranulation.

## Materials and Methods

### Ethics Statement

All guinea pig work was conducted according to the German guidelines for animal care and welfare (Deutsches Tierschutzgesetz) and approved by the Bavarian state ethics committee (Regierung Oberbayern, which serves as the Institutional Care and Use Committee for the Technische Universität München) according to §4 and §11 Deutsches Tierschutzgesetz under the reference number 32-568-2.

All procedures for rat experiments are according to UK Home Office guidelines and with ethical approval from the University of Sheffield (UK) (PPL 40/3119).

### Ca^++^ and Voltage Sensitive Dye Imaging in the Enteric Nervous System

All methods and techniques have been previously described in detail [13], [14].

### Primary Culture of Enteric Neurons

Experiments were performed on primary cultures of myenteric neurons from guinea pig (Dunkin-Hartley) small intestine. Male animals (Harlan, Borchen, Germany) were killed by cervical dislocation and exsanguination according to the German guidelines for animal care and welfare after §4 of Deutsches Tierschutzgesetz. Approximately 10–15 cm of the small intestine orally to the ileocecal sphincter was quickly removed and rinsed with sterile, ice cold Krebs solution which was also used for the following preparation steps (in mM: 1.2 MgCl_2_, 2.5 CaCl_2_, 1.2 NaH_2_PO_4_, 120.9 NaCl, 14.4 NaHCO_3_, 11.5 Glucose, 5.9 KCl; gassed with 95% O_2_, 5% CO_2_; pH 7.4). The tissue was cut into segments of 10–20 mm length under a sterile bench. The myenteric plexus together with longitudinal muscle and serosa was stripped and samples from 10 segments were washed three times in fresh solution and collected in a small glass bottle with 4.5 ml Krebs solution. The samples were cut to small pieces with scissors and solutions of protease type I (8 U/ml, Gibco, Karlsruhe, Germany), collagenase type II (516.2 U/ml, Gibco) and bovine serum albumin (BSA fraction V 0.37%, Serva, Heidelberg, Germany) were added. After 20–30 min of incubation at 37°C tissue samples were washed three times with ice cold Krebs solution. Thereafter the pellet was suspended in 10 ml cold culture medium (M199 GlutaMAX, Gibco/Invitrogen, Darmstadt, Germany) with 1% Penicillin/Streptomycin (Gibco/Invitrogen), 30 mM glucose, 50 ng/ml mouse nerve growth factor 7S (Alomone Labs, Jerusalem, Israel) and 10% fetal bovine serum (Gibco/Invitrogen) and placed on ice. To collect ganglia for cultivation the suspension was put into a petri dish and placed on a microscope (Leica DMIL, 4x objective with phase contrast). Individual ganglia were picked with a pipette (100 µl tip) up to a total volume of approximately 100 µl. This volume was used to inoculate a culture dish (µ-dish 35mm with ibiTreat-coating, Ibidi, Martinsried, Germany). After incubation for 1–2 hrs at 5% CO_2_, 37°C, 100% humidity, 1 ml neuronal medium was added and ganglia were maintained in culture for at least 7 days. Medium was changed every 2–3 days and supplied with 2 µM cytosine β-D-arabinofuranoside (Sigma) to suppress the proliferation of other cell types.

### Staining Procedures

We used Di-8-ANEPPS as a voltage sensitive dye and Fluo-4 AM as a calcium reporter dye (both dyes from Invitrogen, Karlsruhe, Germany). Stock solutions of Di-8-ANEPPS (Invitrogen, 15 mM in a 50%/50% mixture of DMSO “extra dry” (Acros Organics, Geel, Belgium) and Pluronic F-127 (20% in DMSO, Invitrogen) and Fluo-4 AM (Invitrogen, 1 mM in DMSO) were stored at −20°C. Prior to the experiment the stock solutions were thawed and diluted to final concentrations (Di-8-ANEPPS: 10 µM, Fluo-4 AM: 10 µM) with HEPES buffer (in mM: 1 MgCl_2_, 2 CaCl_2_, 150 NaCl, 10 HEPES, 10 Glucose, 5 KCl). For Fluo-4 AM 0.5–1.25 mM Probenecid was added to the HEPES buffer for incubation of the dye and to the superfused solution during the experiment. After taking a culture dish from the incubator the culture medium was gently removed with a pipette. Then 1 ml of the Di-8-ANEPPS solution was added and the dish was left 15 min or for the Fluo-4 AM solution 20 min for incubation at room temperature in the dark. The staining solution was removed and the preparation was mounted in a special self-made Plexiglas recording chamber and continuously perfused during the experiment with 37°C HEPES buffer.

Some preliminary experiments were performed on freshly dissected whole mount preparations from guinea pig submucous plexus. These preparations were loaded with Fluo-4 AM for 2 hrs followed by 1 hr washout period.

### Neuroimaging

The signals were recorded with a CCD camera (frame rates up to 2 kHz, 80×80 pixels; (NeuroCCD-SMQ, RedShirt Imaging, Decatur, USA) and analysed with the NeuroCCD software (RedShirt Imaging). The fluorescence filterset for Di-8-ANEPPS consisted of a modified Cy3 filter (excitation: HQ545/30X, dichroic: DM565, emission BA580IF, AHF Analysentechnik, Tübingen, Germany). For the Fluo-4 AM we used a FITC filterset (excitation: HC482/35, dichroic: BS506, emission: HC536/40, AHF Analysentechnik). During the experiment, the submucous plexus preparations or enteric neuron cultures were continuously perfused with Krebs (containing in mM: 117 NaCl, 4.7 KCl, 1.2 MgCl_2__*_ 6H_2_O, 1.2 NaH_2_PO_4_, 25 NaHCO_3_, 2.5 CaCl_2 *_ 2H_2_O and 11 glucose) or HEPES buffer (containing in mM: 1 MgCl_2_, 2 CaCl_2_, 150 NaCl, 5 KCl, 10 Glucose, 10 HEPES), respectively.

### Recordings from Visceral Afferents

Mesenteric afferent discharge was recorded electrophysiologically from pentobarbitone anaesthetized Hooded Lister rats (60 mg/kg followed by 0.5 to 1 mg/kg/hr) according to UK Home Office guidelines and with ethical approval from the University of Sheffield. Experiments were performed on naïve animals and animals that had been sensitized to chicken egg albumin (EA; Sigma, Poole, Dorset, UK) as described previously [15]. Mesenteric afferent recordings were performed 10–14 days following sensitization and only animals that demonstrated a functional anaphylactic response at the termination of the study were included for subsequent analysis.

The methodology for afferent recording has also been previously described [15] and essentially involved the preparation of an isolated loop of jejunum through which 1 ml of EA (10 mg/ml) or compound 48/80 (1 mg/ml; Sigma) diluted in normal saline solution could be infused while recording the afferent impulse traffic in a paravascular nerve bundle supplying the segment. After stabilizing the preparation for 30 min compound 48/80 or EA were infused over 15 s with free drainage from the distal end of the loop. This brief period of infusion ensured that the intraluminal pressure and mechanical activation of the afferents had subsided prior to the onset of the chemically evoked response. Administration was repeated at 5 min intervals. In cross desensitization experiments administration of compound 48/80 or EA was repeated after prior desensitization to the other stimulus.

### Primary Cultures of DRG and Nodose Neurons

Mouse dorsal root ganglia (DRG T9-L2) and nodose ganglia were removed and placed in cold Hank′s Balanced Salt Solution (HBSS; pH 7.4; Gibco, Invitrogen, UK). Ganglia were rinsed and dissociated [16]. Briefly whole ganglia were incubated in 4 mg/ml papain (Sigma) which had been previously activated with 0.7 mg/ml cysteine (Sigma) (DRG 20 min, nodose 10 min, 37°C). The papain solution was then removed and replaced with HBSS containing 4 mg/ml collagenase (Sigma) and 4.7 mg/ml dispase (Sigma) (DRG 20 min, nodose 10 min, 37°C). The collagenase/dispase solution was removed and the ganglia were rinsed to stop the enzymatic reaction with DMEM:F12 tissue culture medium (Gibco, Invitrogen, UK) with 10% fetal bovine serum (Gibco). The ganglia were then placed in 0.5 ml tissue culture media and triturated 10 times using a plastic Pasteur pipette until the solution appeared cloudy. Cells were then plated on matrigel coated coverslips and incubated for 24 hrs at 37°C. DRGs and nodose were loaded with 4 µM Fura-2 AM for 30 min at 37°C in the dark. The coverslips were transferred to the bath chamber and were continuously superfused with HEPES buffer (containing in mM: 135 NaCl, 5 KCl, 2 CaCl_2_, 1 MgCl_2_, 10 HEPES and 10 glucose) for 30 min at room temperature. For application of compound 48/80 the coverslip was superfused with buffer containing compound 48/80 (10 µg/ml).

### Drug Application in Studies on Enteric Neurons

Compound 48/80 was purchased from Sigma (Schnelldorf, Germany) and dissolved in HEPES buffer at a stock concentration of 1 mg/ml. The pH of the compound 48/80 solution was 7.4. Compound 48/80 (100 µg/ml and 10 µg/ml) was applied to enteric neurons by pressure ejection from fine tipped glass pipettes (0.1–3 s pulse duration), which resulted in about a final tenfold dilution of the pipette content [17]. Therefore, the final concentration of compound 48/80 at the level of the neurons was about 10 µg/ml and 1 µg/ml and thereby at the highest concentration around the EC50 of compound 48/80 to induce mast cell mediator release [10].

Nicotine (Sigma) was applied to the neurons by pressure ejection at a concentration of 100 µM. Nicotine was chosen as it evoked Ca^++^ transients in almost all cultured enteric neurons [13]. We perfused cultured enteric neurons with the H_1_ antagonist pyrilamin (1 µM) and H_2_ antagonist ranitidine (10 µM) (both from Sigma) in order to block histamine mediated activation of enteric neurons. As a mast cell stabilizer we used cromolyn sodium salt (100 µM) (Sigma) [18], [19]. It was perfused for 20 min and its effect on the compound 48/80 and nicotine evoked enteric nerve activation was assessed.

### Immunohistochemistry

Immediately after the neuroimaging experiments, the cultured neurons were fixed overnight at 4°C in a solution containing 4% paraformaldehyde and 0.2% picric acid in 0.1mol/l phosphate buffer and then washed (3×10 min) in phosphate buffer. The cells were permeabilized with phosphate-buffered saline (PBS) containing 0.5% Triton X-100, 0.1% NaN_3_ and 4% horse serum for 1 h at room temperature. This was followed by 12 hrs incubation with the primary antibody and 1.5 hrs with the secondary antibody in the permeabilization solution. As primary antibodies we used rabbit anti-c-kit (c-kit; 1∶100; Oncogene; Cambridge, MA, USA) rabbit anti-calbindin (Calb; 1∶1000; Chemicon, Limburg, Germany) and sheep anti-human protein gene product 9.5 (PGP9.5; 1∶20000, The Binding Site, Birmingham, UK). As secondary antibodies we used donkey anti-rabbit conjugated to 7-amino-4-indodicarbocyanin (Cy5; 1∶200; Dianova, Hamburg, Germany), donkey anti-rabbit conjugated to carbocyanin (Cy2; 1∶500; Dianova, Hamburg, Germany), donkey anti-sheep conjugated to 7-amino-4-indodicarbocyanin (Cy5; 1∶500; Dianova, Hamburg, Germany) and donkey anti-sheep conjugated to Indocarbocyanin (Cy3; 1∶500; Dianova). After immunohistochemistry the cultured neurons were treated with 1 mM CuSO_4_ in 50mM ammonium acetate buffer to reduce autofluorescence of the cells. Finally the cells were washed in PBS and mounted with a solution of PBS (pH 7.0) and 0.1% NaN_3_ containing 80% glycerol. The preparations were examined with an epifluorescence microscope (Olympus, Japan) equipped with appropriate filter blocks. Images were acquired with a video camera connected to a computer and controlled by Scion image software (Scion Corp., Frederick, MD, USA).

For toluidine blue stainings fixed cultured neurons were incubated with a toluidine blue solution containing 0.1% toluidine blue, 7% ethanol and 0.9% sodium chloride in distilled water (pH 2.0). Cultured neurons were washed with distilled water and thereafter incubated with the toluidine blue solution for 2 min. Finally, after 3 washes with distilled water, the culture dishes were mounted as described above. Mast cells appear in violet/red against a blue background. Both toluidine blue and c-kit reliably label mast cells [20], [21], [5].

### Data Analysis and Statistics

For experiments in enteric neurons Ca^++^ measurements were performed as single trial recordings and Di-8-ANEPPS measurements as three 3 s long trials with intervals of 6 s. Data from camera pixels belonging to individual neurons was averaged and displayed as % change of resting fluorescence (ΔF/F). For [Ca^++^]_i_ measurements we identified the maximal value during the measurement period. Signals were only included if their amplitude was at least twice the background noise signal. Normally distributed data are given as means ± standard deviations while non-normally distributed data are reported as median values with the 25% and 75% quartiles given in brackets. The response of compound 48/80 and nicotine before and after cromolyn was compared with a Wilcoxon signed rank test.

For the recording of visceral afferents the whole nerve mesenteric afferent firing was quantified as imp/sec during a 15 s period at the height of the response. The response profile was evaluated from peristimulus rate histograms. Data are presented as mean values ± standard error of the mean with N = number of animals and statistical significance determined by paired and unpaired Student *t* test as appropriate.

For experiments in DRG and nodose neurons Fura-2 AM ratios were calculated by monitoring the fluorescence signal elicited from excitation at 350 nm and 380 nm wavelengths using an LED UV illuminator. Coverslips were superfused with HEPES buffer containing compound 48/80 (10 µg/ml) for 2 min. [Ca^++^]_i_ was expressed as a percentage of the maximum level obtained in the presence of the calcium ionophore, ionomycin (5 µM) and plotted as mean values ± standard error of the mean.

*P* values of <0.05 were considered significant.

## Results

### Effects of Compound 48/80 in vivo

**Figure 1:**
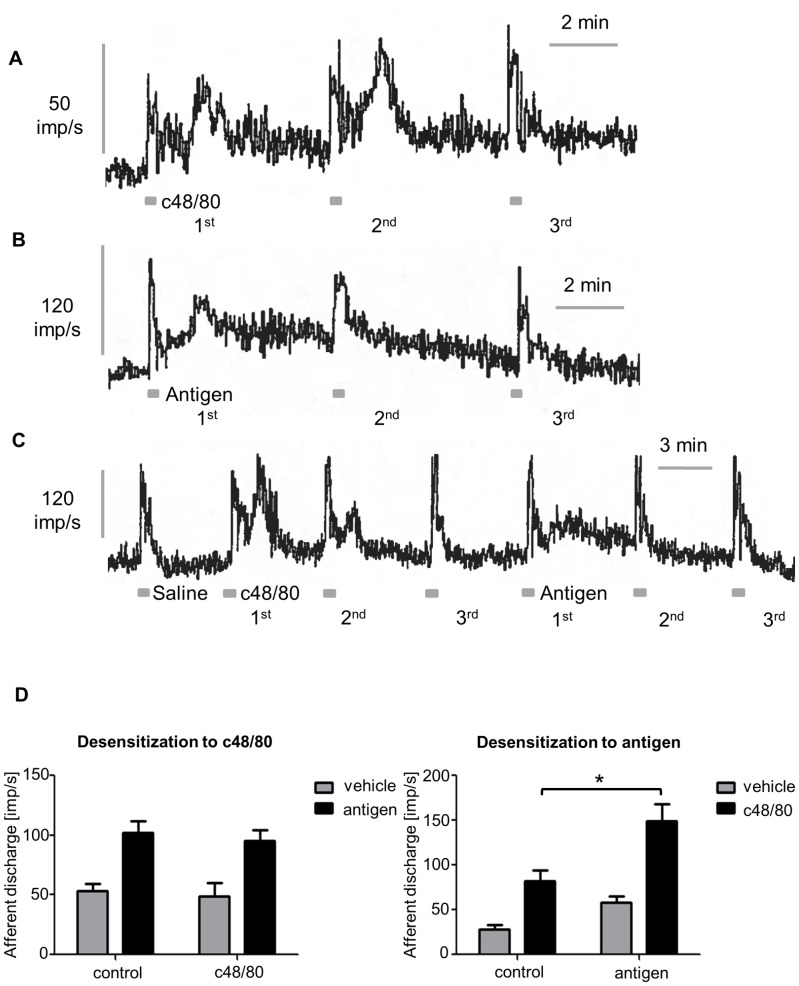
Compound 48/80 (c48/80) evoked activation of visceral afferents *in vivo*. A Jejunal afferent discharge stimulated by c48/80 administered intraluminally. A second administration 5 min later evoked a similar afferent response but subsequent administrations are completely desensitized, despite similar mechanosensitivity during the luminal perfusions. **B** In sensitized animals intraluminal application of the antigen egg albumin (EA) evoked a response similar to that observed with c48/80 and which too rapidly desensitized so that subsequent antigen failed to evoke a response. **C** Following desensitization to c48/80, intraluminal antigen still evoked a response similar to that in naïve control animals. When the order of administration was reversed and the response to antigen was desensitized, c48/80 was still able to evoke a response **D** illustrates mean data from these cross-desensitization experiments. The left panel shows the afferent response to saline or antigen in sensitized animals that had received pretreatment with vehicle (saline, N = 11) or c48/80 (N = 5). The panel on the right shows similar data for the response to vehicle (saline) or c48/80 in naïve control animals (N = 9) or sensitized animals following desensitization to antigen (N = 6). The response to c48/80 was significantly augmented after desensitization to antigen (P<0.01).

Compound 48/80 applied intraluminally evoked a marked increase in discharge after a mean latency of 54 s which recovered towards baseline after about 1 min (Figure 1A). Discharge increased from a baseline of 28±5 to peak at 82±12 imp/s. (*P*<0.001; N = 9). A second administration 5 min later evoked a similar afferent response but subsequent administrations were completely desensitized. In sensitized animals intraluminal egg albumen evoked a response similar to that observed with compound 48/80 and which too rapidly desensitized so that subsequent antigen failed to evoke a response (Figure 1B). In cross-desensitization experiments the response to antigen persisted after compound 48/80 (Figure 1C) and vice versa responses to compound 48/80 persisted after EA. Indeed the response to compound 48/80 was significantly augmented after desensitization to antigen (P<0.01). The quantification of this data is shown in Figure 1D. The lack of cross-desensitization indicates that antigen-evoked mast cell degranulation and compound 48/80 act through different mechanisms.

### Effects of Compound 48/80 on Enteric Neurons

In preliminary experiments which were originally aimed to selectively stimulate mast cells in submucous plexus preparations we locally applied compound 48/80 onto submucous ganglia for several seconds via pressure application. This caused an unexpected Ca^++^ signal in 51% of 32 neurons (5 ganglia, 1 animal). Since the submucous plexus preparation contained numerous mast cells in close proximity of ganglia, the Ca^++^ signal in nerves could have been secondary to the release of neuroactive compound from mast cells.

**Figure 2:**
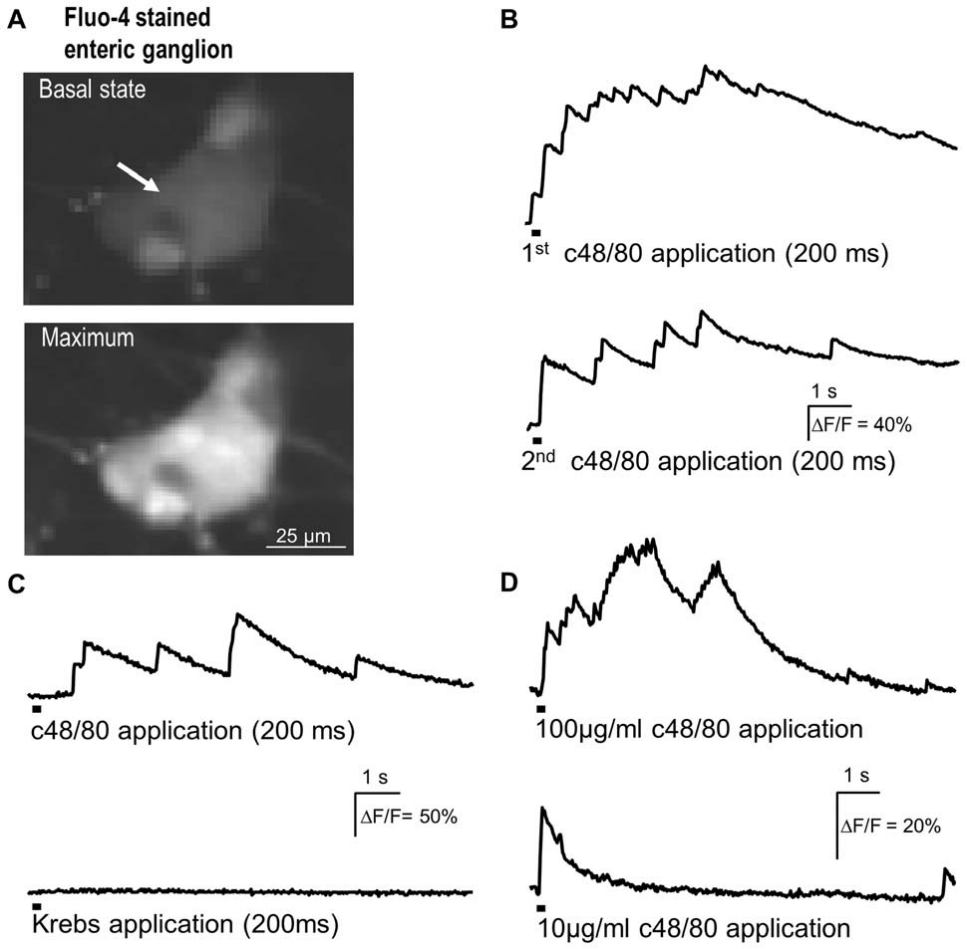
Compound 48/80 (c48/80) evoked Ca^++^ transients in primary cultured enteric neurons. A shows two images of a Fluo-4 AM stained ganglion before c48/80 application and at the time of the maximal response. **B** illustrates the Ca^++^ signal of the neuron marked by an arrow in A. Two consecutive c48/80 applications (marked by the bars below the traces) evoked comparable responses. In this ganglion all 8 neurons responded to c48/80. **C** confirms that responses to c48/80 application were not caused by activation of mechanosensors because spritz application of Krebs solution using the same application parameters had no effect. **D** illustrates that c48/80 evoked [Ca^++^]_i_ increase is concentration dependent.

**Figure 3:**
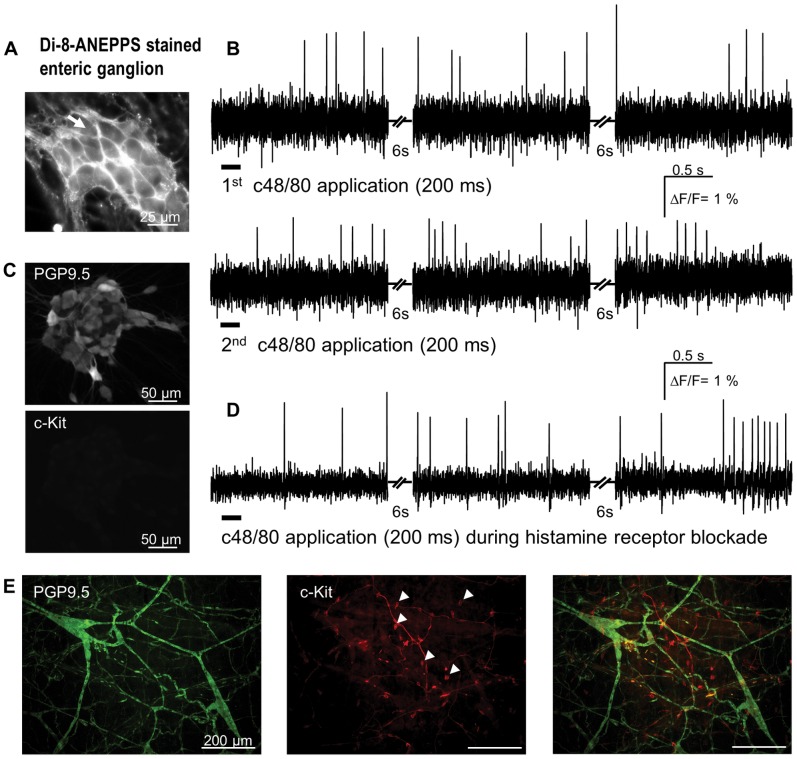
Compound 48/80 (c48/80) evoked spike discharge in primary cultured enteric neurons. A Di-8-ANEPPS stained ganglion; the dye incorporates into the outer membrane revealing the outline of individual neuronal cell bodies. **B** illustrates action potential discharge in response to two consecutive c48/80 applications (marked by bars below the traces) from the neuron marked by white arrow in A. The traces show responses during three recording periods with non-recording periods of 6 s in between. In this ganglion 17 of 19 neurons responded to c48/80. **C** PGP9.5-positive cultured enteric neurons. Lack of c-kit immunoreactivity demonstrated lack of mast cells in the culture. **D** demonstrates that c48/80 application still evokes spikes in cultures treated with a combination of the H_1_ and H_2_ blockers pyrilamine (1 µM) and ranitidine (10 µM), respectively. **E** shows green PGP9.5-positive neurons in a whole mount preparation of the guinea pig submucous plexus (left panel) and red c-kit-positive round and smooth mast cells (some marked by white triangles in center panel), which are morphologically distinct from the spindle shaped interstitial cells of Cajal. The right panel shows the merged image of the two stainings.

We therefore studied the nerve activating actions of compound 48/80 in primary cultured enteric neurons. Indeed, spritz application of compound 48/80 evoked Ca^++^ transients in 80 [67/100] % of 402 neurons (38 clusters, 5 animals) studied (Figure 2). These cultures contained no mast cells demonstrated by the negative c-kit (Figure 3) and toluidine blue staining. The median maximal increase was 52 [24/96]% ΔF/F. This was a substantial increase but smaller than observed after application of nicotine (142 [80/243]% ΔF/F; p≤0.001). The time to reach the maximum of the Ca^++^ transient was significantly faster for nicotine than compound 48/80 (1.8 [0.7/2.4] s versus 3.8 [1.6/6.9] s; p≤0.001). Furthermore as a control the same Krebs solution as superfused was in the same way as compound 48/80 applied onto the neurons to rule out mechanical stimulation. Spritz application of Krebs did not induce any Ca^++^ transients (Figure 2). We found no evidence that enteric glia cells respond to compound 48/80, because all responding cells in a cluster were also activated by nicotine and were PGP9.5-immunoreactive. None of the PGP9.5-negative cells showed Ca^++^ transients in response to compound 48/80 (73 neurons, 9 clusters, 2 animals).

In cultured enteric neurons stained with the voltage sensitive dye Di-8-ANEPPS, spritz application of compound 48/80 for 0.1 to 3 s resulted in long lasting discharge of action potentials in previously quiescent neurons. Compound 48/80 activated 95 [75/100]% of 124 neurons (6 clusters, 1 animal) (Figure 3). Prolongation of the spritz duration resulted in gradual increase in spike frequency and an earlier onset of spike discharge. With a 0.1, 0.2 and 3 s spritz application the first spike appeared after 11.1 [10.4/12.7] s, 2.5 [2.0/9.3] s and 1.5 [0.9/2.2] s, respectively. The spike discharge rate increased from 0.2 [0.1/0.6] Hz to 1.9 [0.7/2.8] Hz after 0.1 s and 0.2 s spritz application, respectively.

The nerve activating effect of compound 48/80 was concentration dependent. Lowering the concentration in the spritz from 100 µg/ml to 10 µg/ml significantly decreased the proportion of responding neurons (65±27% versus 34±37%; p = 0.026) as well as the increase of [Ca^++^]_i_ (31 [14/70]% ΔF/F versus 20 [11/33]% ΔF/F; p = 0.031); data based on 93 neurons (7 clusters, 2 animals) (Figure 2).

Compound 48/80 induced spike discharge was not changed after blockade of histamine H_1_ and H_2_ receptors which are responsible for histamine mediated excitation of guinea pig enteric neurons [5] (Figure 3). During blockade of histamine actions 0.2 s application of 100 µg/ml compound 48/80 activated a median of 100 [58/100]% of 26 neurons (3 clusters, 1 animal). They fired at a frequency of 0.9 [0.5/1.7] Hz.

The compound 48/80 induced rise in Ca^++^ was significantly faster in onset than the appearance of the first action potential suggesting that Ca^++^ increase proceeded and likely triggered spike discharge. The Ca^++^ signal started to raise 0.7 [0.3/1.1] s after 0.2 s application. The compound 48/80 induced Ca^++^ increase as well as the enhanced spike discharge was reproducible (Figure 2, 3).

The mast cell stabilizer cromolyn significantly attenuated the response to 100 µg/ml compound 48/80. Cromolyn decreased both the number of responding neurons (82±18% versus 66±25%; p = 0.032) as well as the amplitude of the Ca^++^ transients (37 [25/73]% ΔF/F versus 27 [14/48]% ΔF/F; p = 0.002; 111 neurons, 10 clusters, 3 animals). It is noteworthy, that cromolyn also attenuated the response to nicotine evoked Ca^++^ transients (189 [130/270]% ΔF/F versus 130 [91/169]% ΔF/F; p≤0.001; 64 neurons, 6 clusters, 2 animals).

### Characterization of the Enteric Neurons Responding to Compound 48/80

Calbindin may be used as a marker for neurons with a slow after spike hyperpolarization (AH) [22]. In 73 neurons of 9 clusters from 2 animals we could show that sensitivity for compound 48/80 is not related to Calbindin immunoreactivity and hence not a hallmark of AH neurons. Compound 48/80 activated 74% of all neurons but only 7% of these were labeled by Calbindin and PGP9.5. Of the remaining neurons which did not respond to compound 48/80, 21% were immunoreactive for Calbindin and PGP9.5 whereas 79% were only PGP9.5 immunoreactive.

### Effects of Compound 48/80 on Sensory Neurons

**Figure 4:**
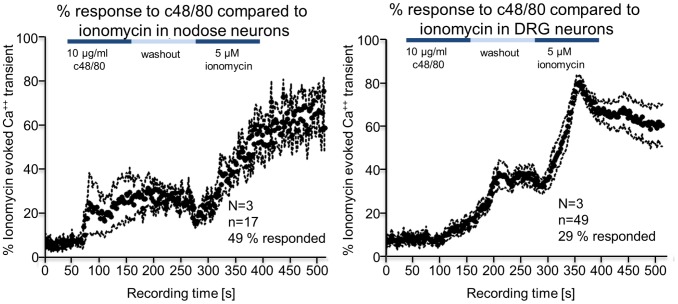
Compound 48/80 (c48/80) evoked Ca^++^ transients in isolated DRG and nodose ganglion cells in culture. In both cases c48/80 evoked a calcium signal that was about 40% of the maximum response to the calcium ionophore, ionomycin. The response of nodose and DRG neurons to c48/80 was different in two respects, first in the proportion of cells responding and in the time course of the onset of the response. More nodose neurons responded than DRG and the onset of the response was quicker.

Calcium imaging was performed on isolated DRG and nodose ganglion cells in culture. In both neuronal cell types compound 48/80 evoked an increased calcium signal with a peak response of 38.7±3.8% in DRG and 31.5±7.6% in nodose neurons when normalised to the maximum response to the calcium ionophore, ionomycin (Figure 4). The response of nodose and DRGs to compound 48/80 is different in 2 respects. It is first different in the proportion of cells responding and in the time course of the onset of the response. More nodose neurons (49%) responded than DRG (29%) and the onset of the response was quicker.

## Discussion

Numerous studies using compound 48/80 in tissue or animal models contributed to the generally accepted view that the observed effects are through mast cell degranulation. Strikingly, we could only identify three studies which confirmed this mode of action in isolated mast cells; these studies demonstrated that compound 48/80 degranulates mast cells and that this effect is reversed by mast cell stabilizers [23]–[25]. However, our findings showed that compound 48/80 directly activated enteric nerves and visceral afferents via a mechanism that is independent of mast cells. This conclusion is based upon 3 sets of observations. First, in vivo activation of mesenteric afferents persists after antigen-induced mast cell mediated hypersensitivity responses have desensitized. In this model the mast cell mediated response is attenuated by pharmacological blockade of mast cell mediators, particularly histamine and 5-HT [15]. Secondly, in calcium imaging studies using isolated DRG and nodose neurons in culture we found a robust response to compound 48/80. Thirdly, submucosal neurons in whole mount preparations and cultured myenteric neurons respond rapidly to compound 48/80 and since the cultures did not contain mast cells, based on the negative c-kit and toluidine blue staining, this has to be mediated via a mechanism independent of mediator release from mast cells. Furthermore, we demonstrated that excitation in cultured enteric neurons by compound 48/80 was not altered by histamine receptor antagonists which would be expected if mast cells were involved.

The most likely explanation is that this is a direct effect of compound 48/80 on neuronal excitability. Indeed Heppner and Fiekers, (1992) [12] described effects of compound 48/80 on sympathetic neurotransmission in the bullfrog that included increased excitability of dissociated sympathetic neurons.

Moreover, compound 48/80 did not change firing rate of neurons in the cortex and hippocampus, where no mast cells are present, but activated thalamic neurons [26], [27]. Circumstantial evidence suggested involvement of mast cells in the hypothalamus because consecutive morphological analysis showed mast cell degranulation. It is possible that different molecular properties of brain neurons may influence their responsiveness to compound 48/80. Incubation of mouse hypothalamic brain slices with compound 48/80 (10−100 µg/ml) caused release of histamine from non-mast cell compartments, most likely nerves, as well as release of the neurotransmitters dopamine, noradrenaline and serotonin [28].

The effect of compound 48/80 was consistent across 3 different populations of neurons: enteric, DRG and nodose. However, not every neuron in these preparations responded to compound 48/80 suggesting that this is not a general (toxic) effect on excitable cells. In this respect the concentration of compound 48/80 used in our in vitro studies (1−10 µg/ml) is well within the range used in cell culture models by others. In addition compound 48/80 excited two populations of enteric neurons based on the finding that both Calbindin-positive as well as Calbindin-negative neurons were activated. 50% of Calbindin and PGP9.5 immunoreactive neurons responded to compound 48/80 and were likely AH-neurons [22]. Of the remaining PGP9.5 positive but Calbindin negative neurons, 77% were excited by compound 48/80.

The pattern of response to compound 48/80 was also different between these 3 neuronal populations. In enteric neurons the onset occurred with a few seconds of spritz application while in sensory neurons the onset was delayed following superfusion. This difference could reflect the time course of drug delivery. With spritz application the concentration will peak rapidly and thereafter decline while with superfusion the drug concentration will rise slowly as the buffer in the bath is replaced by buffer containing drug. Nevertheless, there were clear differences between DRG and nodose in the time course of activation despite identical drug delivery. This might suggest that phenotypic difference in individual neurons within these 3 neuronal populations might determine their sensitivity to compound 48/80.

It was beyond the scope of this study to identify the mode of action of compound 48/80 to activate neurons. It may involve modulation of ionic conductance as suggested by Heppner and Fiekers, (1992) [12]. Alternatively, mechanisms involving cell metabolism are considered to trigger mast cell degranulation [29] and may also apply in neurons. The action of compound 48/80 is commonly attributed to a direct activation of the G(i/o) class of G proteins [30] or activation of phospholipase D [31]. Recently, it has been shown that compound 48/80 targets mas related genes, a family of G protein coupled receptors also known as sensory neuron specific receptors [32]. Further studies would be necessary to determine the down-stream effects of compound 48/80 but may provide mechanistic understanding relevant to drug development for treating conditions like IBS in which an interaction between nerves and mast cells is considered to be an etiological factor [33]. Interestingly, the mast cell stabilizer cromolyn attenuated the compound 48/80 evoked activation in enteric neurons. We may rule out stabilization of mast cells as a possible reason because our primary cultures were devoid of mast cells. We rather suggest that this effect is due to unspecific actions of mast cell stabilizers based on our finding that cromolyn also attenuated the response to nicotine. It has been demonstrated that chromones, including cromolyn, eliminated voltage dependent Ca^++^ currents in smooth muscle cells [34]. While interference with Ca^++^ mobilization is involved in the mast cell stabilizing actions of cromolyn, this effect may also account for the decrease in compound 48/80 and nicotine evoked Ca^++^ transients in enteric neurons.

In a previous study in which cross-talk between mast cells and DRG neurons was examined, the supernatant from mast cells stimulated with compound 48/80 evoked a neuronal response while compound 48/80 alone had no effect [10]. How this observation differs from the current findings is unclear. However, mast cells were incubated with compound 48/80 for 1 hr while the neurons were exposed for seconds following application of an aliquot of compound 48/80 to the bath in which it would have been rapidly diluted. In our cultures the neurons were superfused at a known concentration of compound 48/80 for 2 min providing a more prolonged exposure. Moreover, the time course of activation in DRGs was very much slower than that for nodose neurons and the calcium signal was still rising at the end of the 2 min exposure. In the De Jonge et al (2004) [10] study it is therefore possible that exposure to compound 48/80 was insufficient in concentration and duration to evoke a direct effect while allowing the more rapid responses to mast cell mediators evoked by long-term exposure to compound 48/80 to be distinguished.

In summary our data clearly demonstrate effects of compound 48/80 and cromolyn that are independent of mast cells. This raises a cautionary note when interpreting the actions of compound 48/80 as well as mast cell stabilizers as mast cell specific. Functional changes measured in model systems that contain neurons must consider a direct neural action of compound 48/80 to increase neuronal excitability in addition to mast cell degranulation.
